# Emergency Management of Extensive Spinal Epidural Hematoma Causing Medullary Compression After Single-Level Anterior Cervical Discectomy and Fusion (ACDF): A Novel Segmental Decompression and Catheter Irrigation Strategy

**DOI:** 10.7759/cureus.86426

**Published:** 2025-06-20

**Authors:** Chao Y Gu, Wang Chen, Hao Yan, Ling Zhu

**Affiliations:** 1 Spine Surgery, The Affiliated Hospital of Wuhan Physical Education University, Wuhan, CHN

**Keywords:** catheter irrigation, postoperative cervical spine surgery, postoperative spinal epidural hematoma (seh), spine, surgical management options

## Abstract

Here, we present a retrospective analysis of a 48-year-old male patient who developed a rare complication of extensive spinal epidural hematoma (SEH) with spinal cord and medullary compression following single-level anterior cervical discectomy and fusion (ACDF), manifesting as acute dyspnea, coma, and quadriplegia. The emergency treatment entailed anterior segmental decompression under general anesthesia, removal of internal fixation devices, and targeted catheter irrigation to evacuate the hematoma spanning from C1 to C7. Postoperatively, the patient demonstrated remarkable neurological recovery, with the American Spinal Injury Association (ASIA) impairment grade improving from preoperative grade A (complete paralysis) to grade E (normal function), and was discharged after uneventful rehabilitation. This case highlights that despite the low incidence of SEH after cervical spine surgery, prompt intervention with segmental decompression combined with catheter irrigation offers a novel, low-trauma, and efficient strategy for managing life-threatening medullary compression, achieving rapid hematoma clearance and favorable functional outcomes.

## Introduction

Anterior cervical discectomy and fusion (ACDF) is the most commonly used surgical approach for treating cervical spondylotic myelopathy or radiculopathy [1]. Spinal epidural hematoma (SEH) is a common complication after ACDF surgery. Most cases are asymptomatic, and generally, only the presence of a hematoma can be detected by imaging, which does not affect the surgical outcome of patients [2]; however, a small number of patients present with symptomatic SEH. The disease progresses rapidly, is difficult to observe, and has a relatively high mortality rate [3]. Although there are clinical reports on this, most of them focus on analyzing the risk factors for developing this disease, with few reports on how to manage it. In particular, there are very few reports on the treatment methods for long-segment SEH, and there is almost no reference for the emergency treatment of medulla oblongata compression. A case of medullary compression due to a long spinal epidural hematoma spanning C1 to C7 following a single-segment cervical spine anterior cervical discectomy and fusion (ACDF) operation has been reported for the first time. After decompression and catheter flushing, the patient was stabilized, ultimately recovered, and was discharged from the hospital. The details of the case are presented below.

## Case presentation

Materials and methods

General Information

The patient was a 48-year-old male. He initially presented with neck pain two years ago, which was exacerbated upon neck flexion. No specific treatment was administered at that time. Ten days before admission, the neck pain intensified, accompanied by pain in the left forearm and the radial aspect of the elbow joint, along with numbness in the left index and middle fingers, with a Visual Analogue Scale (VAS) score of five. The pain and numbness were severe and could be alleviated to some extent by elevating the left upper limb. Conservative symptomatic treatment proved ineffective. Upon physical examination, the patient was found to have a relatively short neck with a straightened physiological curvature. The neck muscles were rigid. His neck demonstrated a normal range of motion in flexion and extension. The Lhermitte, Spurling, and Eaton tests were all positive, while the left Hoffmann sign was negative. The biceps and triceps reflexes were active, with a muscle strength rating of 4 + for both. No obvious pathological signs were detected in the lower limbs. Japanese Orthopaedic Association Score (JOA) was 13. The patient had a history of hypertension, which was well-controlled through administered medications. His blood pressure upon admission was measured at 120/70 mmHg, and he had no history of taking non-steroidal anti-inflammatory drugs. After admission, comprehensive examinations were performed, including cervical spine radiography (DR), computed tomography (CT), magnetic resonance imaging (MRI), cervical vascular Doppler ultrasound, electrocardiogram, and chest CT. The cervical imaging findings indicated left-sided nerve compression at the C6/7 level, which correlated well with the patient's symptoms and physical examination findings (Figures 2, 3). Pre-operative evaluations were complete, and laboratory tests, including liver and kidney function and coagulation function, were within normal ranges. Given the failure of conservative treatment and at the request of the patient and his family, surgical intervention was deemed appropriate. The patient underwent microscopic C6/7 ACDF surgery under general anesthesia. The operation proceeded smoothly, lasting for 1.2 hours. Adequate hemostasis was achieved during the procedure, and a drain was placed before concluding the operation. The patient regained consciousness and was transferred to the ward in a stable condition.

**Figure 1:**
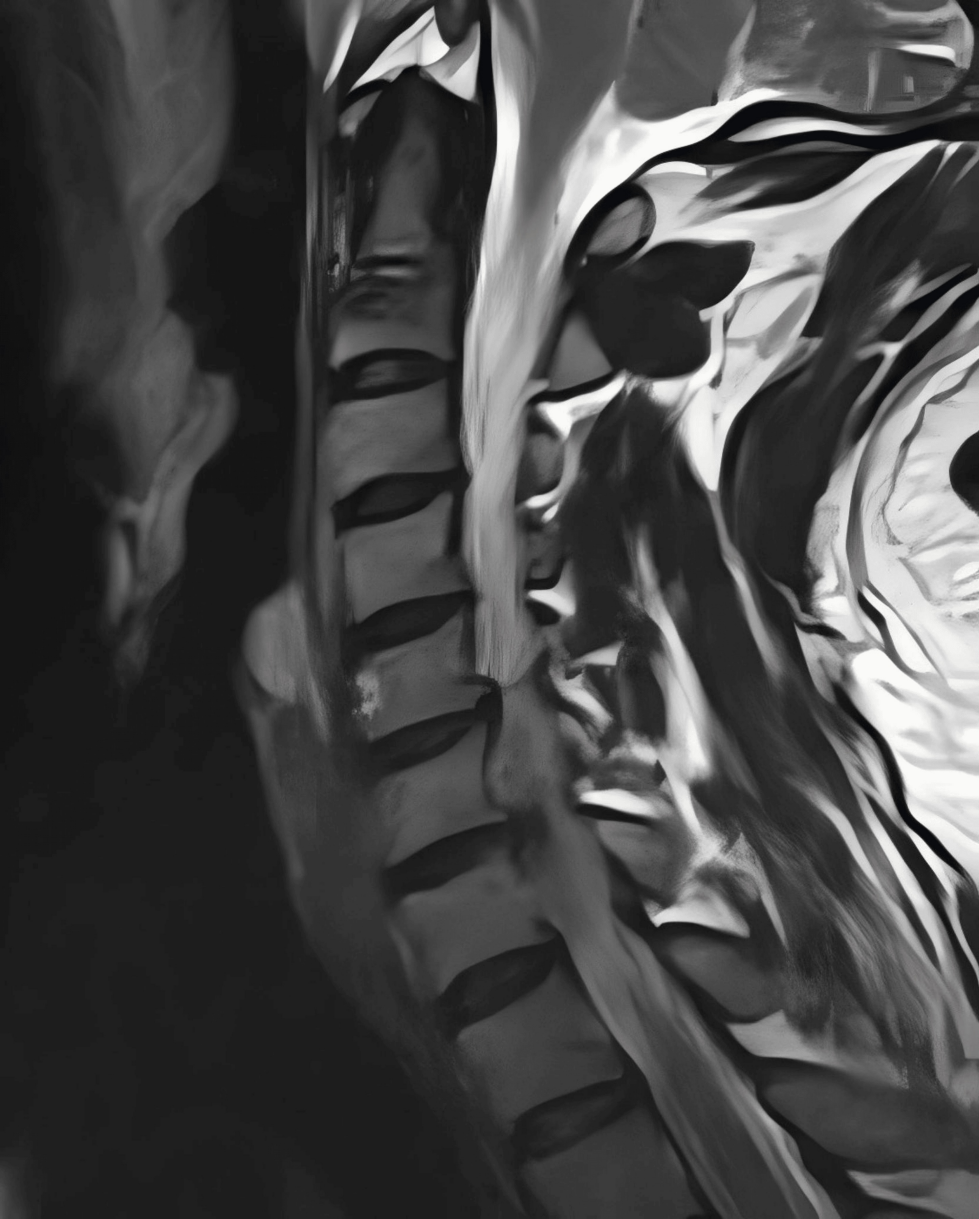
Patient's pre-operative magnetic resonance imaging (MRI) The patient's sagittal MRI shows a herniated disc at C6/7, compressing the nerves at the back

**Figure 2:**
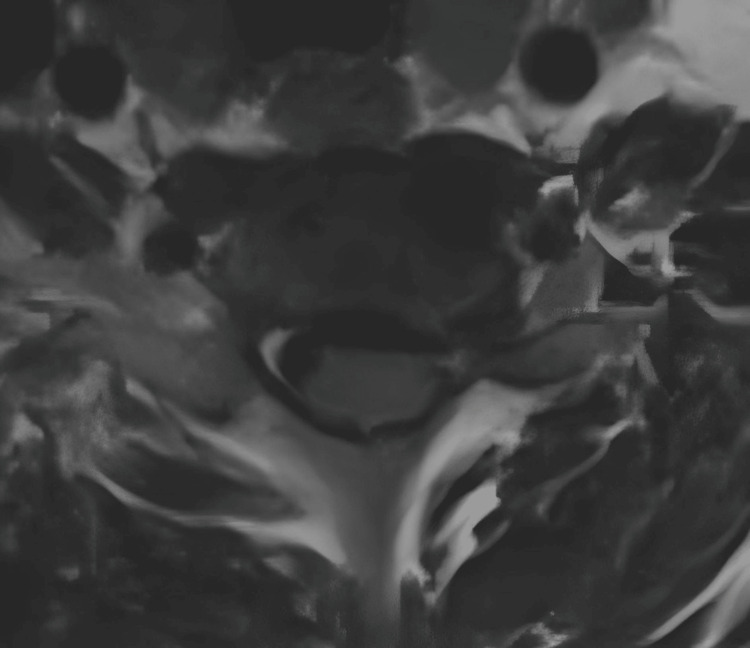
Patient's preoperative magnetic resonance imaging (MRI) The cross - section of the patient's cervical spine MRI shows that the intervertebral disc protrusion compresses the dura mater and nerve roots, especially in the left intervertebral foramen area, which is more obvious.

Clinical Manifestations

**Figure 3:**
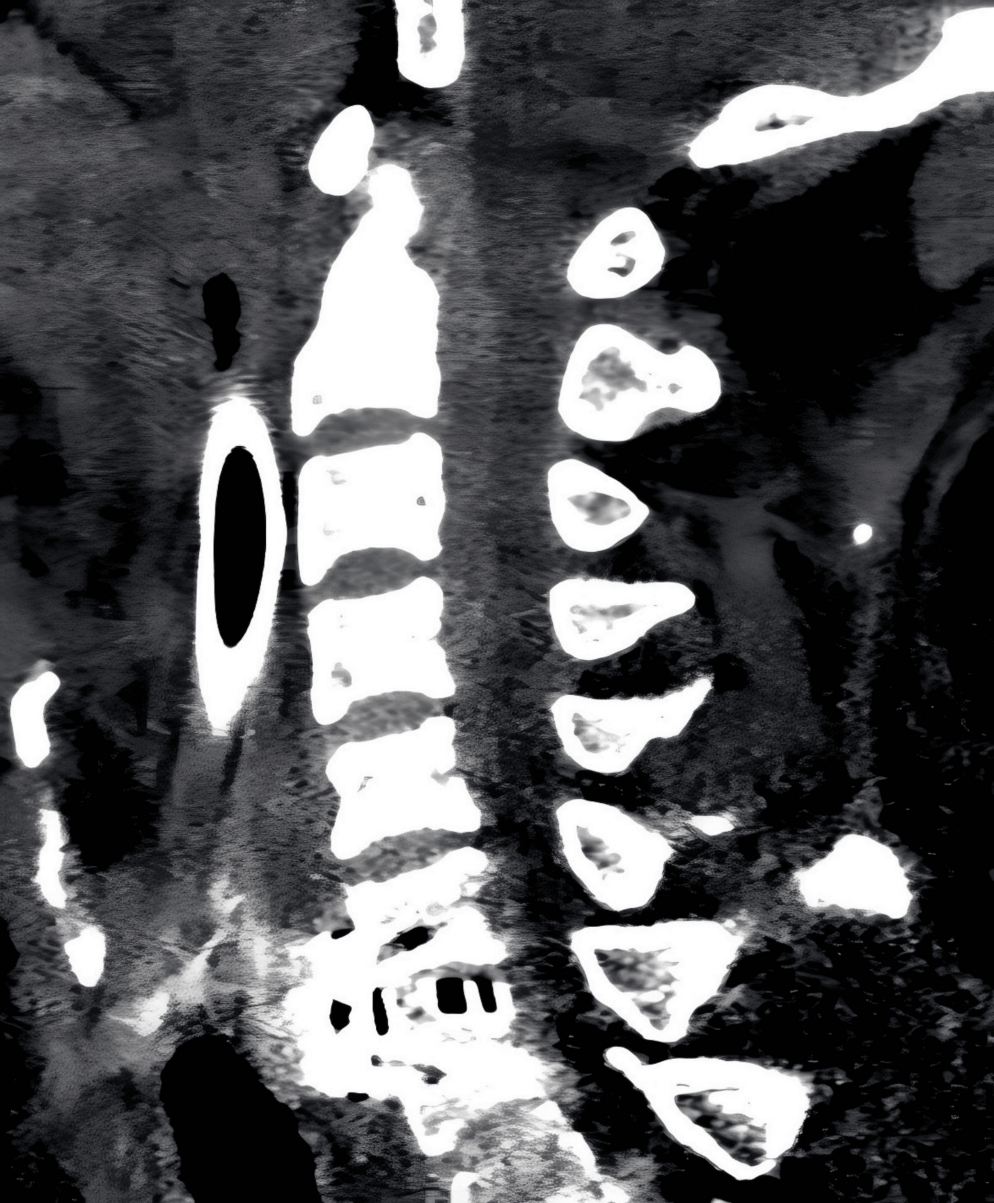
Cervical computed tomography (CT) scan performed emergently after the onset of spinal epidural hematoma (SEH) It reveals an epidural hematoma extending from C1 to C7, with significant compression of the cervicomedullary junction.

Post-operation, upon returning to the ward, the patient exhibited stable vital signs, clear consciousness, and normal motor and sensory functions in all four limbs. The symptoms in the left upper limb were notably alleviated compared to the pre-operative state. The patient was placed on electrocardiogram monitoring, provided with oxygen supplementation, and received symptomatic treatment. Approximately one hour after the operation, the patient complained of pain in the posterior neck. Considering that this might be attributed to maintaining the intraoperative position, continuous observation was initiated. At one hour and 10 minutes post-operation, the patient reported weakness in the left lower limb, making it difficult to lift. A check of the neck revealed unobstructed drainage and normal neck muscle tone. At one hour and 12 minutes post-operation, the patient started to experience chest tightness and dyspnea. The oxygen saturation was measured at 98%, blood pressure at 126/80 mmHg, and respiratory rate at 19 breaths per minute. Just two minutes later, at one hour and 14 minutes post-operation, the patient lost his voice, and the respiratory rate increased. The oxygen saturation dropped slightly to 96%, blood pressure rose to 130/90 mmHg, and the respiratory rate reached 24 breaths per minute. Additionally, the tendon reflexes in all four limbs disappeared, muscle tone became absent, and the Glasgow Coma Scale (GCS) score plummeted to three points (E1V1M1). Subsequently, the patient became unresponsive to verbal stimuli, with neurological decompensation (Table 1). The patient was immediately rushed to the operating room for tracheal intubation and connected to a ventilator. After appropriate adjustments, the vital signs stabilized. Given the patient's sudden deterioration, possible diagnoses of SEH or intracranial hemorrhage were considered. However, due to the tracheal intubation, MRI could not be performed. Instead, cranial and cervical spine CT scans were conducted, which revealed the presence of SEH extending from C1 to C7 (Figure 3).

**Table 1:** Timeline of disease deterioration The process from after the patient's surgical operation to the loss of consciousness

After surgery	1 hour after the operation	1 hour and 10 minutes after the operation	1 hour and 12 minutes after the operation	1 hour and 14 minutes after the operation
Vital signs are stable	Neck pain	Weakness in the left lower limb	Feeling stuffy and having difficulty breathing	Aphonia, rapid breathing, disappearance of tendon reflexes in the extremities, loss of muscle tone, and coma

Treatment Methods

**Figure 4:**
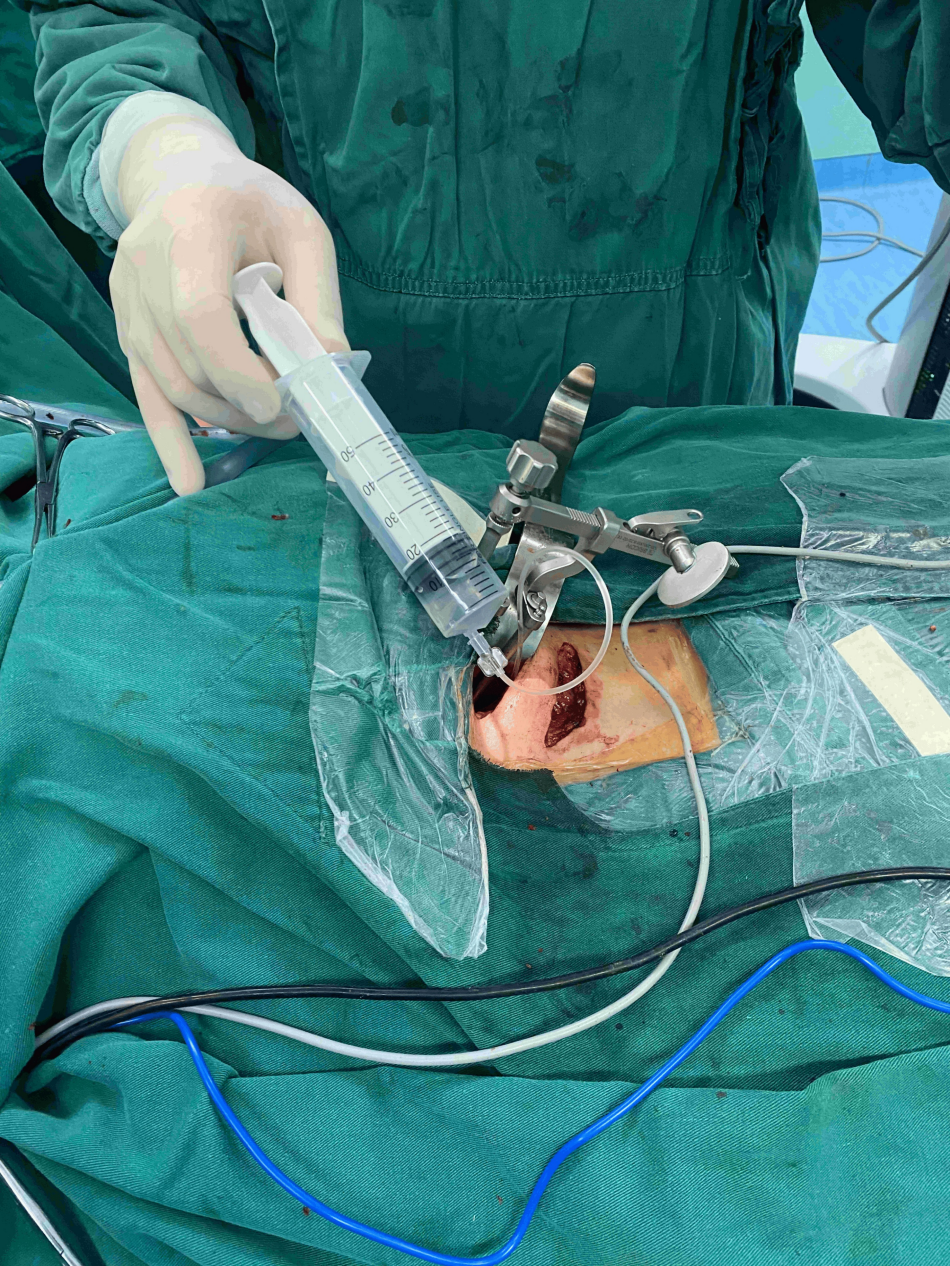
Flush with the syringe connected to the catheter

**Figure 5:**
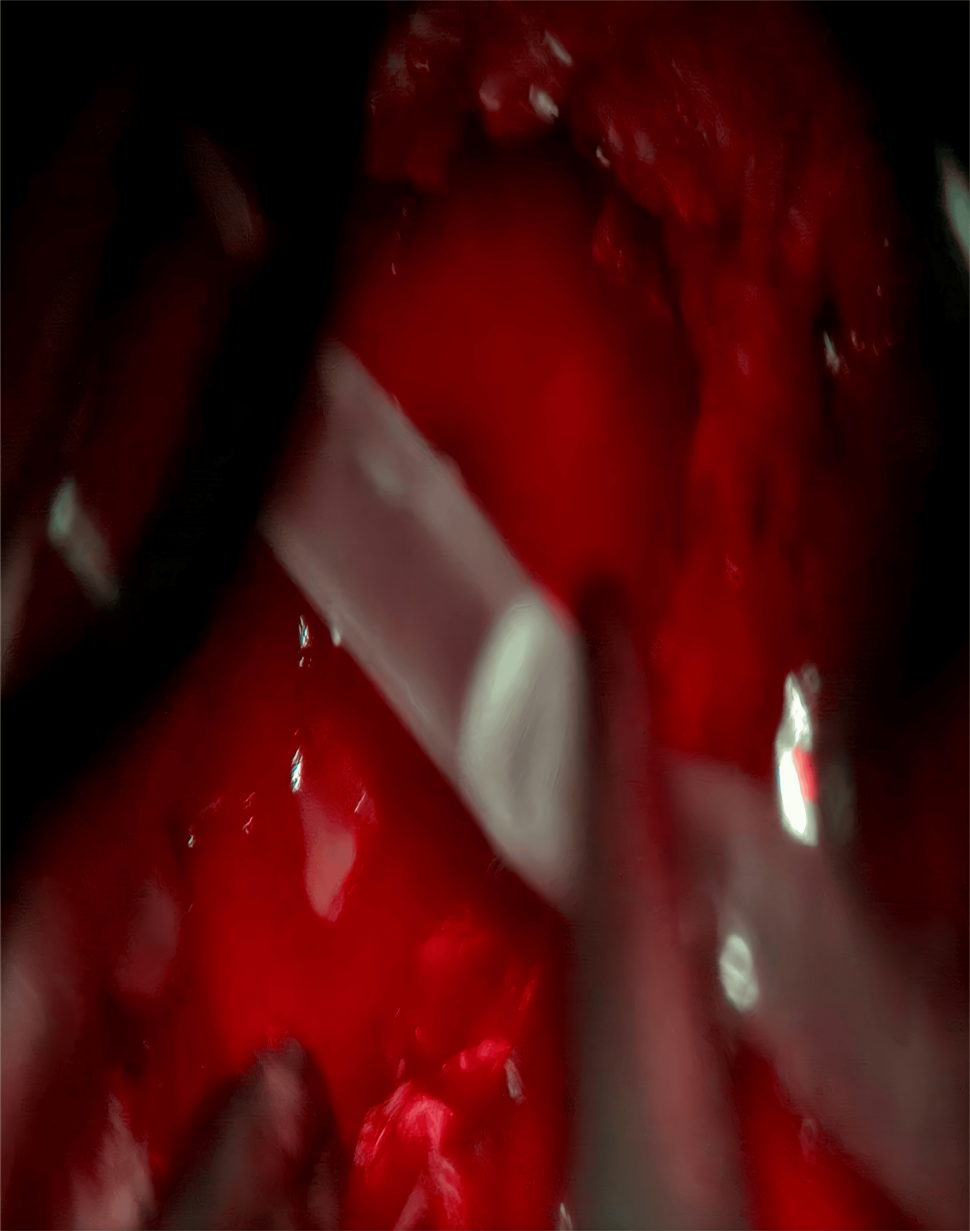
Microscopic examination shows catheter irrigation

Once the patient was under optimal general anesthesia, he was positioned in the supine position. The shoulder and back were elevated with padding, and the cervical spine was fixed in a slightly hyperextended position using a fixation bracket. Intraoperative neurophysiological monitoring, encompassing somatosensory evoked potential and motor evoked potential, was continuously carried out throughout the procedure. The surgical field was routinely disinfected and draped. Instruments such as a high-frequency electrotome, bipolar coagulator, drill, and microscope were set up. First, the surgical sutures were carefully removed to expose the C6/7 intervertebral space. Subsequently, the screws, titanium plate, and intervertebral fusion cage were retrieved. Intra-operatively, blood clots were identified in the epidural space, accompanied by minor bleeding. The clots were meticulously removed, and absorbable hemostatic material (Tecossman) was applied for spot hemostasis. This intervention restored the normal pulsation of the dural sac. Due to the high-lying location of the hematoma, a 4-cm transverse incision was made at the level of the hyoid tubercle. The skin, subcutaneous tissue, and platysma were incised sequentially. The deep cervical fascia was dissected, and the space between the visceral sheath and the vascular nerve sheath was carefully separated until the C3 vertebral body was reached. Vertebral body screws were then implanted into the C3 and C4 vertebral bodies, and a vertebral body distractor was inserted to create space. A sharp-edged knife was used to incise the annulus fibrosus, and a curette was employed to remove the nucleus pulposus tissue until the anterior surface of the posterior longitudinal ligament was exposed. Special attention was paid to ensure the complete removal of osteophytes on the posterior edge of the vertebral body. A microscope was introduced, and the posterior longitudinal ligament was incised under its magnification to further clean the posterior edge of the vertebral body. More blood clots were found in the epidural space and were promptly removed. 50Ml syringe connected to the external anesthesia puncture catheter (Jiangsu Maichuang medical apparatus--brand names of medical devices, F3-1 type, diameter 1.0mm) (Figure 4). The tube was gently inserted upward along the posterior edge of the C3 vertebral body until it reached the C2 vertebral body. Normal saline was then used for flushing (Figure 5). As a result, blood clots were observed flowing out from the C3/4 intervertebral space. After repeated flushing until the effluent fluid was clear and free of clots, the tube was inserted downward along the posterior edge of the C4 vertebral body in a similar fashion until it reached the posterior edge of the C5 vertebral body. Normal saline flushing was repeated, and blood clots were seen emerging from the C6/7 intervertebral space. After thorough flushing until the fluid was clear, the pulsation of the dural sac was restored. Intraoperative neurophysiological monitoring indicated a satisfactory recovery of the patient's motor and sensory functions. Medical-induced bone matrix (Shanghai Xiaobo-brand names of medical devices) was used to fill the cage. A cage was implanted into the C3/4 intervertebral space. An appropriate - length anterior cervical spine titanium plate (4-hole * 16 mm, Zhengtian-brand names of medical devices) was selected and fixed to the C3 and C4 vertebral bodies using four screws (φ4 * 14 mm, Zhengtian-brand names of medical devices), and the titanium plate was securely locked. Subsequently, a Cage (7 * 15 * 12 mm, Zhengtian-brand names of medical devices) was implanted into the C6/7 intervertebral space. Another appropriate - length anterior cervical spine titanium plate (4 - hole * 14 mm, Zhengtian-brand names of medical devices) was fixed to the relevant vertebral bodies with four screws (φ4 * 14 mm, Zhengtian-brand names of medical devices) and locked in place. Fluoroscopy confirmed the optimal positioning of the intervertebral fusion cage and internal fixation devices. After ensuring complete hemostasis, a meticulous count of cotton pads, gauze, and surgical instruments was conducted. A disposable multi-functional negative drainage tube (Guangdong Xianlai S6A-2--brand names of medical devices) was placed in the surgical wound. Finally, the incision was sutured in a step-by-step manner, marking the completion of the operation.

Results

**Figure 6:**
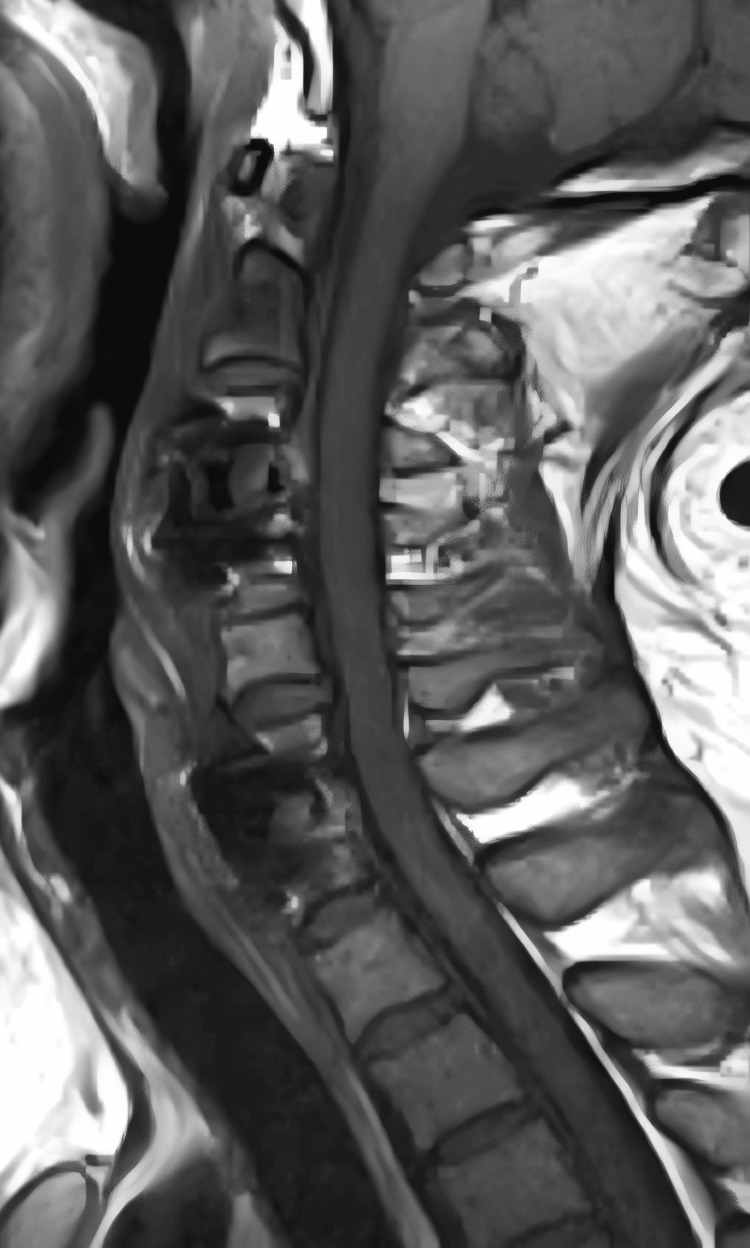
Postoperative MRI Postoperative cervical MRI shows that the hematoma has been completely removed.

The total operative time was two hours and 15 minutes, with intraoperative blood loss of 90 mL. Post-operation, the patient regained consciousness promptly, with normal sensory and motor functions in all four limbs. The vital signs remained stable. Physical examination revealed that the muscle strength of the four limbs had returned to the pre-operative level (4+). The tendon reflexes of the four limbs were normal, neither hyperactive nor weakened. There was no numbness in the saddle area, and both the cremasteric and anal reflexes were intact. The abnormal sensations in the left upper limb had completely resolved, with no remaining pain or numbness. The ASIA impairment scale improved from grade A before surgery to grade E. The patient was administered anti-infection medications and other appropriate symptomatic treatments. Ten days after the operation, all of the patient's physiological indicators returned to normal. VAS was 2, JOA was 17, and the surgical improvement rate is 76%. A follow-up MRI scan showed complete resolution of the hematoma (Figure 6). With no remaining discomfort, the patient was discharged from the hospital after a successful rehabilitation period.

Follow-up

**Figure 7:**
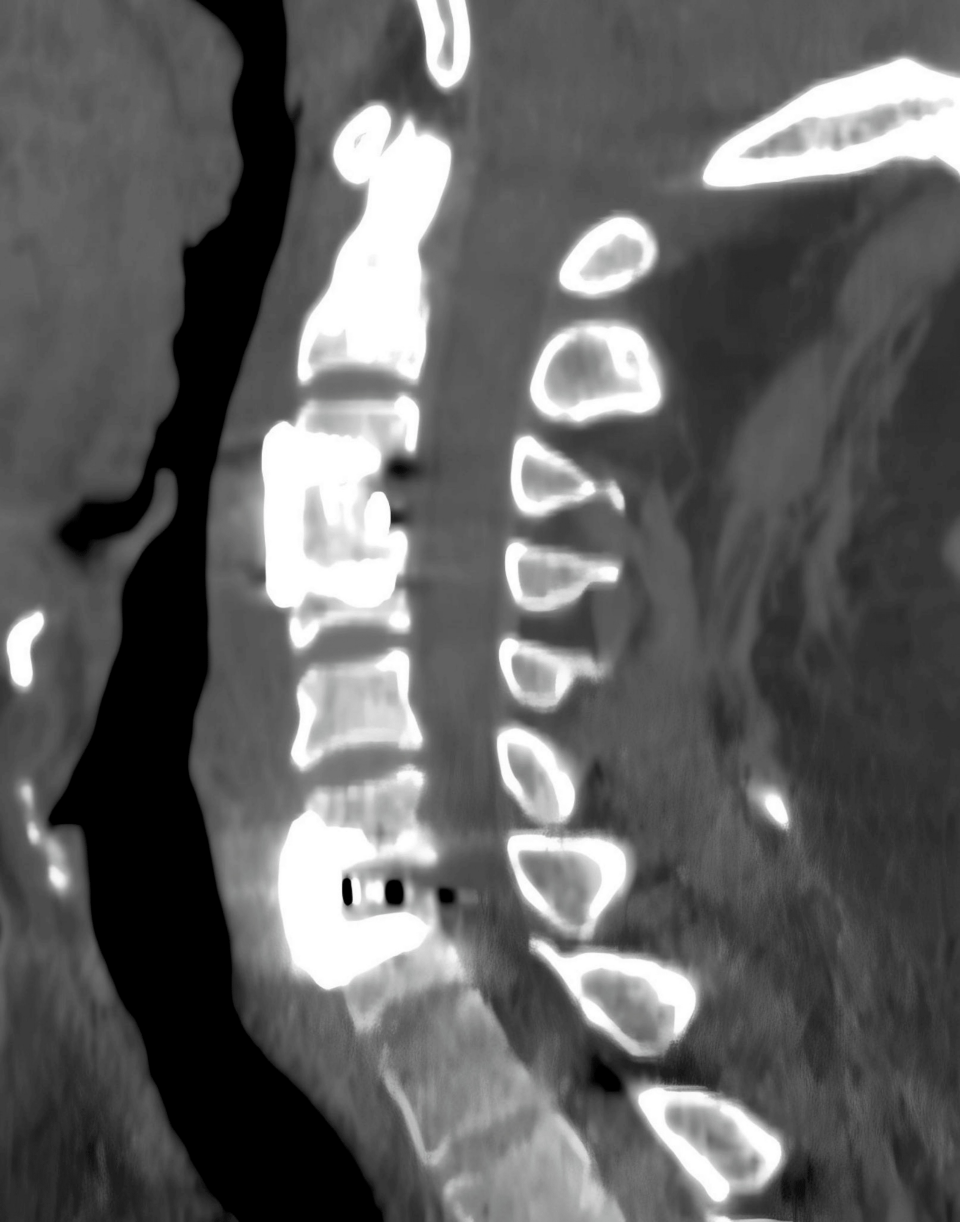
CT scan one month after surgery One month after the surgery, CT scan showed that the cervical hematoma was completely resected and the internal fixation was in a good position.

**Figure 8:**
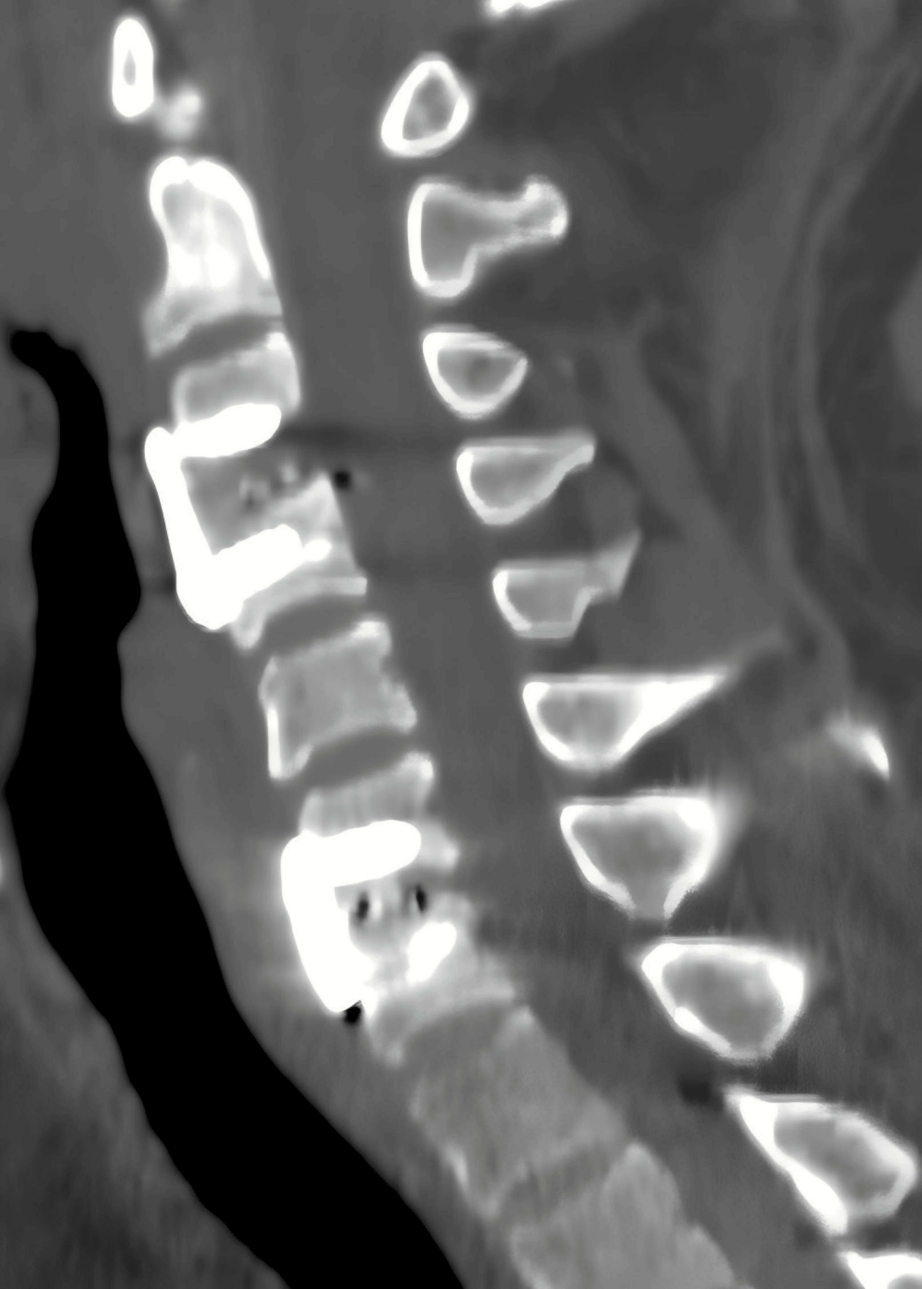
CT scan three months after the surgery Six months after the operation, the CT scan showed no hematoma, the internal fixation was in good position, and fusion had occurred.

One month after discharge, the patient underwent a follow-up CT scan (Figure 7). The results indicated the absence of any visible hematoma, stable spinal alignment, and no displacement of the internal fixation devices. The patient's motor and sensory functions remained normal, with an ASIA impairment scale of grade E, and there were no signs of neurological deficits. During the six-month follow-up (Figure 8), the patient's self-care ability had significantly improved, and he was able to return to work. By the one-year follow-up, the patient's quality of life continued to improve, and he was able to perform his work duties and engage in daily activities without any restrictions.

## Discussion

SEH was first described by Jackson [4]. He reported the case of a 14-year-old female who unfortunately died after cervical spine surgery. He hypothesized that cervical epidural hematoma might be the cause, which was later confirmed by autopsy. This case brought the severity of SEH to the attention of the medical community. In reality, the incidence of symptomatic SEH after cervical spine surgery is relatively low. Research by Schroeder et al. [5] indicated that the re-operation rate for SEH after cervical spine surgery ranged from 0.2% to 0.24%. Aono et al. [6] reported that among 1376 patients who underwent cervical spine surgery, 466 of them had anterior-approach surgery, and only one patient developed SEH (0.21%). Xia et al. [7] reported that in their center, out of 18,220 patients who underwent cervical spine surgery, 10,514 had anterior-approach procedures, and 13 of them developed SEH (0.12%). In our hospital, over the past five years, 295 anterior cervical interbody fusion surgeries were performed, and only two cases of symptomatic SEH occurred, with only one case involving long-segment hematoma compression of the spinal cord. These statistics demonstrate that the incidence of SEH is not high. However, once SEH occurs, the hematoma can directly compress the spinal cord or trachea, leading to severe neurological deficits and even respiratory failure, which can be life-threatening. After the onset of SEH, the patient's condition often deteriorates rapidly. Jankowski et al. [8] suggested that SEH commonly occurs within six hours after surgery, while Amiri et al. [9] reported that it can occur within four hours. In this particular case, the patient developed symptoms just one hour after surgery, and it took only 15 minutes from the onset of discomfort to the loss of consciousness. Therefore, vigilant and timely observation of the patient's postoperative condition is crucial for the early detection of SEH. Healthcare providers should not be complacent simply because of the low incidence of SEH or normal vital signs shown on electrocardiogram monitoring. In this case, the patient's vital signs were relatively normal before the onset of consciousness disorders, which could easily mislead the medical team. Thus, any signs such as shoulder and back pain, abnormal muscle strength in the four limbs, respiratory abnormalities, or voice changes should prompt medical staff to consider the possibility of SEH. In addition, when SEH is suspected, cervical spine MRI should be performed immediately, as it is regarded as the "gold standard" for diagnosing SEH [10]. In certain situations where MRI is not feasible, such as in this patient due to tracheal intubation, CT can be used as an alternative diagnostic tool. Once SEH is confirmed, urgent cervical spine hematoma evacuation is essential. Although limb disorders and changes in consciousness can be caused by other conditions like cerebral infarction and intracerebral hemorrhage, emergency anterior cervical spine hematoma evacuation or exploration is a necessary step. In extreme cases, when SEH is strongly suspected and time is of the essence to save the patient's life, it may be justifiable to proceed with emergency exploration without waiting for imaging results.

The low incidence of SEH is counterbalanced by its rapid progression and high mortality rate. Timely and appropriate treatment is of utmost importance. Although there are existing clinical reports on SEH, most of them are retrospective studies that mainly focus on identifying the risk factors for its development. There is a lack of in-depth research on specific surgical strategies after the diagnosis of SEH, especially for long-segment SEH. In 2003, the Belgian doctor Pol Hans reported a case [11] of a patient who developed a long-segment SEH from C3 to T1 after C6/7 ACDF surgery. In 2021, Italian doctor Roberta Morace [12] reported a case of cervical 2-thoracic 1, but there was little reference for reports of SEH rising to the medulla oblongata, affecting the respiratory center and requiring emergency treatment. Epstein et al. [13] emphasized that once SEH is diagnosed, emergency surgical decompression should be carried out within six hours to optimize the prognosis of neurological function. Yin et al. [14] demonstrated through research that performing hematoma evacuation within 24 hours after the onset of SEH can significantly contribute to the recovery of neurological function. However, there is currently no consensus on the optimal method for hematoma clearance. Clinically, the standard approach for treating SEH often involves reopening the original surgical incision, removing the anterior titanium plate and cage, achieving hemostasis, and evacuating the hematoma. If the hematoma has a large extent, partial vertebral body resection via the anterior approach may be necessary for decompression. In cases where the anterior approach is not sufficient, posterior approach surgeries such as posterior laminectomy or posterior open-door laminoplasty are considered. Huang Xianhua et al. [15] studied 768 cases of anterior cervical spine surgery. Among the five patients who developed SEH after surgery and underwent surgical treatment, four had anterior cervical spine hematoma evacuation, and one had a combined anterior cervical spine hematoma evacuation and posterior open-door laminoplasty. The case of cervical 2-thoracic 1SEH reported by Italian doctor Roberta Morace [12] in 2021 adopts the method of fine catheter insertion and flushing, but this method cannot confirm whether the inserted catheter can reach the predetermined area, whether the insertion is too deep will cause spinal cord injury, and the saline flushed by the long segment can only flow out from the insertion direction. There is a possibility that the pressure is too large, or the flushing water and blood clots cannot completely flow out. Therefore, we take the removal of the original titanium plate and the cage first. After the thin flexible tube is connected to the syringe, the upper and lower vertebral bodies are flushed first, and then the ACDF approach is taken at the high SEH level to enter the vertebral canal. After removing the high blood clot, the thin, flexible tube of the syringe is inserted into the lower vertebral body for flushing. Because the lower position has cleared the hematoma, a low-pressure area is formed. When the upper position is flushed, the water and blood in the intervertebral space flow out, thus avoiding the cutting of the vertebral body, without the need for posterior decompression, and the surgery time and trauma are greatly reduced. In this case, the MRI hematoma was completely cleared after treatment according to this method, and the effect is worthy of recognition. Therefore, this method can provide a new idea for long-segment SEH surgery.

The occurrence of SEH is multifactorial. Awad et al. [16] demonstrated that pre-operative long-term use of nonsteroidal anti-inflammatory drugs (NSAIDs) increases the risk of postoperative SEH. Goldstein et al. [17], in a 10-year single-institution retrospective study on posterior cervical spine surgery, found that postoperative use of NSAIDs is an independent risk factor for SEH. This is because NSAIDs have antiplatelet effects. They can inhibit cyclooxygenase-1 (COX-1), preventing platelet aggregation and causing coagulation disorders, which may lead to increased bleeding. Moreover, some NSAIDs can also inhibit cyclooxygenase-2 (COX-2), resulting in the overproduction of prostacyclin (PGI2). PGI2 can damage the arterial vascular wall and cause abnormal coagulation, further increasing the risk of postoperative SEH. Additionally, Kishiya et al. [18] reported that patients with continuous ossification exhibit a bleeding tendency and significant intraoperative blood loss, thereby increasing SEH risk. O'Neil et al. [19] conducted a retrospective study on 2375 anterior cervical spine surgeries and identified ossification of the posterior longitudinal ligament (OPLL) as a risk factor for postoperative SEH. There are also reports suggesting that SEH is related to the number of surgical segments. Amiri et al. [9] reported that multi-segment surgeries are an independent risk factor for SEH, and Kou et al. [20] also indicated that multi-segment surgeries are an important risk factor for SEH. Hypertension is another risk factor for SEH after cervical spine surgery. Kao et al. [21] believed that hypertension can increase blood viscosity, leading to thrombus formation. Blood accumulation that cannot flow out may eventually form a hematoma. Yin et al. [14] found that hypertension can elevate the intravascular pressure in the spinal canal, causing bleeding in the spinal canal venous plexus. If the bleeding is severe enough to exceed the cerebrospinal fluid pressure in the spinal canal, continuous bleeding may occur. There are also reports suggesting that SEH is related to factors such as the patient's age, body weight, coagulation function, intraoperative operation techniques, and operation time. Therefore, it is necessary to comprehensively evaluate the patient's condition before surgery, formulate personalized surgical plans, avoid the use of NSAIDs as much as possible before surgery, minimize multi-segment operations, optimize intraoperative techniques, shorten the operation time, and closely monitor blood pressure and coagulation function after surgery to reduce the incidence of SEH and ensure the safety and effectiveness of surgery. In this case, the patient was a middle-aged male with normal blood pressure on admission, no history of taking NSAIDs before surgery, no obvious OPLL, no coagulation disorders, and only received a single-segment surgery. The operation was performed with great care under the microscope, and sufficient hemostasis was achieved. The blood pressure was well-controlled after surgery. However, SEH still occurred, indicating that even if various risk factors are comprehensively controlled, we still need to be vigilant about the impact of individual differences on postoperative complications. We should not assume that the absence of known SEH risk factors means there will be no problems. It is necessary to further refine the pre-operative assessment and postoperative management strategies.

This case is a single-case report, lacking large-sample statistical analysis. Therefore, the universality of the research results needs to be further verified. In addition, the long-term efficacy and potential complications of the patient after surgery still require long-term follow-up and observation. The catheter flushing method used in this case showed obvious advantages in treating acute epidural hematomas during the operation. However, there are still many aspects that need further exploration, such as whether there are contraindications for the use of the flushing catheter, for example, whether it is applicable in cases of osteophytes on the posterior edge of the vertebral body or spinal canal stenosis. Moreover, quantitative studies on the water pressure, water volume, and insertion depth during flushing are also needed.

## Conclusions

This study introduces a new segmental decompression combined with a catheter irrigation strategy for the treatment of extensive spinal epidural hematoma (SEH) with medullary compression after single-level anterior cervical discectomy and fusion (ACDF). A 48-year-old male patient developed postoperative SEH extending from C1 to C7, leading to rapid neurological deterioration. Urgent anterior segmental decompression and catheter irrigation achieved complete hematoma clearance, and the ASIA impairment scale improved from grade A to grade E. This surgical procedure can completely remove the hematoma, avoiding the risk of residual blood clots compressing the nerves after surgery, which may cause numbness or pain in the residual limbs. This minimally invasive surgical procedure causes less trauma to the body, has a short operation time, less bleeding, and a fast recovery. Compared with traditional surgical procedures such as vertebrectomy, it avoids the related complications caused by long-term postoperative hospitalization. This minimally invasive method shows high safety and effectiveness, providing a new strategy for the emergency treatment of SEH. Early detection and timely intervention are crucial for optimizing neurological outcomes.
